# Designing and Construction of a Cloning Vector Containing *mpt64* Gene of *Mycobacterium tuberculosis*

**Published:** 2018-03

**Authors:** Hosna Zare, Ehsan Aryan, Shadi Alami, Atieh Yaghoubi, Roghayeh Teimourpour, Zahra Meshkat

**Affiliations:** 1 Antimicrobial Resistance Research Center, Mashhad University of Medical Sciences, Mashhad, Iran; 2 Department of Microbiology and Virology, Faculty of Medicine, Mashhad University of Medical Sciences, Mashhad, Iran; 3 Department of Microbiology, School of Medicine, Ardabil University of Medical Sciences, Ardabil, Iran

**Keywords:** *Mycobacterium tuberculosis*, DNA vaccine, cloning vector, *mpt64*

## Abstract

**Background:**

Tuberculosis caused by *Mycobacterium tuberculosis (M. tuberculosis)*, remains as one of the leading causes of deaths worldwide, with nearly two million death cases annually. BCG (Bacille Calmette-Guerin) continues to be the most widely used vaccine in the world, but the protective immunity differs in different parts of the world. Accordingly, new strategies including DNA vaccines are essentially needed. This study was aimed to design and construct a cloning vector containing *mpt64* gene of *M. tuberculosis*.

**Materials and Methods:**

*M. tuberculosis* H37Rv was cultured on Lowenstein Jensen medium, and genomic DNA was extracted. The *mpt64* gene was amplified by PCR using designed specific primers. After the digestion of *mpt64* and pcDNA3.1 (+) by *Bam*HI and *Eco*RI restriction enzymes, the *mpt64* fragment was ligated into the digested vector using *T4 DNA ligase* enzyme. Then, the recombinant vector was transformed into competent *Escherichia coli* (*E. coli)* TOP10 strain. To confirm the colonies of transformed bacteria, antibiotic resistance, colony-PCR, restriction enzyme digestion and DNA sequencing were used.

**Results:**

To confirm the clones, colony-PCR using *mpt64* specific primers was performed and the fragment of 718 bp was observed by gel electrophoresis. Clones were also verified by restriction enzyme digestion using *Bam*HI and *Eco*RI restriction enzymes and the 718 bp fragment was observed. Furthermore, results of DNA sequencing showed 100% homology with the *mpt64* fragment of H37Rv in GenBank.

**Conclusion:**

In this study, the *mpt64* fragment was successfully cloned in pcDNA3.1 (+) vector. This construct can be used in future studies as a DNA vaccine in animal models to induce immune system responses.

## INTRODUCTION

Tuberculosis (TB) is a major global health problem caused by *Mycobacterium tuberculosis* (*M. tuberculosis*) (MTB). According to latest report of World Health Organization (WHO), 6.3 million new cases of TB and 1.7 million deaths were reported in 2016 (1,2). The *Mycobacterium bovis* (*M. bovis)* Bacillus Calmette–Guerin (BCG) is the current vaccine of TB which has been included in Immunization Programs since 1974 (3). BCG provides variable protective efficacies ranging from 0 to 80% in different parts of the world and it has a low level of protection against pulmonary TB (4). The variability in BCG's protective efficacy may be resulted from strain variations in BCG preparations and environmental mycobacteria (5). For these reasons, new TB vaccines must be studied. Currently, new vaccines for TB are being developed such as recombinant BCG, subunit, and DNA vaccines (6,7).

Cell wall and secreted proteins of *M. tuberculosis*, the main antigens which are recognized by host immune system, are the candidates for developing DNA vaccines and this type of vaccines have been used to induce immune responses or as boosters after BCG vaccination (8,9). DNA vaccines encoded epitopes are expressed through major histocompatibility complex (MHC) molecules. Next, helper and cytotoxic T cells recognize the MHCI-antigen complex and strong responses of CD4+ (Th1) and CD8+ (CTL) can be induced by DNA vaccines against *M. tuberculosis* (10). Use of these antigens for the diagnosis of both active and latent TB is suggested in recent studies. The Regions of Differences (RD) encoded proteins are among such antigens. Some of these regions of differences which encode antigens are present in the genome of *M. tuberculosis*, *M. bovis,* and *Mycobacterium africanum* (*M. africanum*), but absent in all BCG substrains genome (11).

ESAT-6 (Rv3875), CFP10 (Rv3874), MPT64 (Rv1980c), and CFP21 (Rv1984c) have been considered as the main immunodominant antigens which are encoded by RD1 and RD2 of *M. tuberculosis*, respectively (12). MPT64 is one of the best-characterized antigens in RD2 (the second absent region from the original BCG strains) and a major secreted protein in the early culture filtrate of *M. tuberculosis* that accounts for about 8% of the total culture filtrate protein. This protein consists of 228 amino acids with molecular weight of 24.8 kDa that has superoxide dismutase and strong cellular immune activity (13,14). This study was aimed to design and construct a cloning vector containing *mpt64* gene of *M. tuberculosis* strain H37Rv.

## MATERIALS AND METHODS

### DNA Extraction and Polymerase Chain Reaction

*M. tuberculosis* strain H37Rv was cultured on Lowenstein Jensen (LJ) medium and was incubated for six weeks at 37°C. DNA extraction was performed using boiling method (15).

The *mpt64* primers were designed by Gene Runner software and were synthesized by CinnaGen Company of Iran. The *mpt64* gene amplification was performed using the following primers, forward:

5′TATTTCGGATCCACCATGGGACGCATCAAGATCTTCAT-3′

and reverse:

5′-CATATATGAATTCCTAGGCCAGCATCGAGTCGATCGCGGAAC-3′

(*Bam*HI and *Eco*RI restriction sites are underlined). The 25 μl PCR reaction mixture contained 1 μl of 100 ng/μl genomic extracted DNA, 0.5 μl of 10mM dNTPs (CinnaGen, Iran), 2.5 μl of 10× PCR buffer (ParsTous, Iran), 1.5 μl of 25mM MgCl_2_ (ParsTous, Iran), 0.2 μl of 5U/μl *Taq DNA polymerase* (CinnaGen, Iran), and 1.0 μl of each 10 μM primer (CinnaGen, Iran).

The DNA amplification involved initial denaturation at 95°C for 4 min, proceeded by 35 cycles of denaturation at 94°C for 30 sec, annealing at 60°C for 30 sec, extension at 72°C for 45 sec, followed by final extension at 72°C for seven minutes. Then, 100 μL of the PCR product was electrophoresed on 1% agarose gel and the *mpt64* fragment purification was performed using AccuPrep^®^ Gel Purification Kit (Bioneer, Korea).

### Cloning of mpt64 fragment into pcDNA3.1 (+) vector

Both pcDNA3.1 (+) plasmid and PCR product were digested by *Bam*HI and *Eco*RI restriction enzymes (Thermo Scientific, Germany) by the reaction of 20 μL purified PCR product, 10 μL of 10× Tango Buffer, 2 μL *Bam*HI (5 U/μL), 2 μL *Eco*RI (5 U/μL), and deuterium-depleted water (DDW) up to final volume of 50 μL for PCR product enzymatic digestion. Also, the reaction of 5 μL pcDNA3.1 (+), 4 μL 10× Tango Buffer, 1 μL *Bam*HI (5 U/μL), 1 μL *Eco*RI (5 U/μL), and DDW up to final volume of 20 μL was prepared for plasmid enzymatic digestion. The digested products were electrophoresed and purified by AccuPrep^®^ Gel Purification Kit (Bioneer, Korea).

The digested *mpt64* PCR product was ligated into the digested vector using *T4 DNA ligase* enzyme in a reaction of 8 μL digested *mpt64* fragment, 14.5 μL digested pcDNA3.1 (+) vector, 2.5 μL *T4 DNA ligase* buffer, 0.2 μL *T4 DNA ligase* (150 U/μL) (Metabion, Germany) and 2 μL of the polyethylene glycol (PEG) 4000 (Thermo Scientific, Germany). After ligation, the competent cells of *Escherichia coli (E. coli)* strain TOP10 was prepared by cold treatment of 0.1 M CaCl_2_/MgCl_2_ solution, and the competency made for acquiring DNA. The prepared *E. coli* competent cells were transformed by recombinant vector using the heat shock method (16). This step also included positive and negative controls for validating the process of competent cell preparation. The transformed bacteria were cultured on LB agar medium containing ampicillin by concentration of 100 μg/mL and were incubated at 37°C overnight. Colonies acquiring the recombinant vector were confirmed using Colony-PCR. The recombinant plasmid was extracted from positive clones using GeneJET Plasmid Miniprep Kit (Thermo Scientific, Germany). The clones were also confirmed using enzymatic digestion by *Bam*HI and *Eco*RI restriction enzymes. For final confirmation, DNA sequencing was carried out by SeqLab co. (Germany) and the results were analyzed by BioEdit software.

## RESULTS

**Figure 1.**
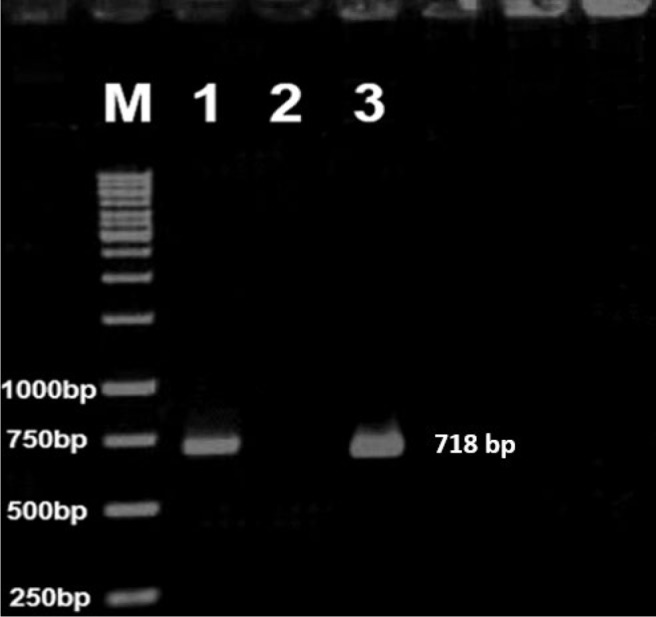
PCR product agarose gel electrophoresis (1% w/v). Lane M: 1 kb DNA Ladder (Thermo Scientific, Germany); Lane 1: positive control of PCR; Lane 2: negative control of PCR; Lane 3: 718 bp PCR product of *mpt64* fragment

In this study, we amplified *M. tuberculosis* strain H37Rv *mpt64* gene by PCR method and fragment of 718 bp was observed by gel electrophoresis (Figure 1). After gel purification of PCR product, single and double digestions were performed on the product.

After digestion of PCR product by *Bam*HI and *Eco*RI, the desired fragment was observed by gel electrophoresis. The pcDNA3.1 (+) vector was also digested with both *Bam*HI and *Eco*RI and a band relating to 5428 bp was observed. Then, *mpt64* fragment was ligated into the pcDNA3.1 (+) vector by *T4 DNA ligase* enzyme. The ligation product was used to transform *E. coli* TOP10 strain using cold 0.1 M CaCl2/MgCl2 solution and transformed bacteria were cultured on ampicillin included LB agar. Colony-PCR was performed using *mpt64* specific primers to confirm insertion of our fragment into the vector and the 718 bp fragment was observed by gel electrophoresis. Purification of recombinant vector was performed by GeneJET Plasmid Miniprep Kit (Thermo Scientific, Germany) and the same fragment was observed in gel electrophoresis for the extracted plasmid PCR by *mpt64* specific primers. Then the recombinant vector was subjected to double digestion with *Bam*HI and *Eco*RI, so the insert cut out and was observed by gel electrophoresis. Final confirmation was based on DNA sequencing of recombinant vector.

## DISCUSSION

The long duration of conventional chemotherapy of TB, together with the increasing incidence of drug-resistant strains and co-infection with HIV, points to an urgent need for new strategies against TB (17). BCG is the only available vaccine and a live attenuated one which has been used since 1921 (18). Despite the worldwide use, it has some limitations such as low level of protection in pulmonary TB. So, TB remains as the major public health problem (18,19). Thus, new effective and reliable vaccines are needed. In this regard, several strategies including DNA vaccines, recombinant BCG vaccines, and subunit vaccines are being developed. They are intended to replace the BCG vaccine or as the boosters after BCG vaccination (20).

The secreted and cell wall proteins of *M. tuberculosis* including MPT64, HSP, Esat-6, Ag85, PstS-3 which recognized by immune system of host, could be the candidates for developing DNA vaccines (13,21).

Mahairas and colleagues demonstrated the *M. tuberculosis* specific genomic regions for the first time in 1996. Their studies showed that three specific regions in the genome of *M. tuberculosis* and *M. bovis*, were absent from BCG genome and were called regions of difference (RD1, RD2, and RD3) (22).

MPT64 (Rv1980c), an important immunodominant antigen with superoxide dismutase activity and a strong cellular immune activity, is encoded by RD2 of *M. tuberculosis* and included in recent vaccine-related studies. This protein has 228 amino acids with the molecular weight of 24.8 kDa (14).

Yu et al, in a study on the Ag85B/MPT64/MPT83 DNA vaccine, showed that the vaccine provides good protection in mice following the exposure to H37Rv antigens. According to their research, the use of DNA vaccine is valuable to reduce the course of treatment (23).

Bai and colleagues expressed the fusion genes of *esat-6* and *mpt64* in *E. coli*. It was observed that the fusion protein of ESAT-6/MPT64 increases Th1 response more than MPT64. It was resulted that the more epitopes detected, the stronger immune responses would occur (24).

Bao et al. has successfully constructed the Ag85A/MPT64 DNA vaccine. Their results showed that animal challenge would produce specific antibodies (13).

Tian et al showed that the DNA vaccine encoding Ag85B and MPT64 increased the IgG titers in immunized C57BL/6 mice to very high level only after the second injection (25).

Fan and colleagues showed that MPT64 could activate RAW264.7 macrophage to induce cytokines significantly (7).

In this study, we cloned *mpt64* fragment into the eukaryotic pcDNA3.1 (+) vector to be used as a DNA vaccine. In further studies, immunization can be considered as a DNA vaccine in laboratory animal models.
